# Effect of Tetrahedrally Coordinated Al on the Surface Acidity of Mg-Al Binary Mixed Oxides

**DOI:** 10.3390/molecules28166072

**Published:** 2023-08-15

**Authors:** Vidya Chandrabose, Taeho Kim, Ji won Park, Sang-Yong Jung, Jae-Min Oh

**Affiliations:** 1 Department of Energy and Materials Engineering, Dongguk University-Seoul, Seoul 04620, Republic of Korea; cbvidya1997@gmail.com (V.C.); taeho0408@naver.com (T.K.); qkrwldnjs324@naver.com (J.w.P.); syjung05@naver.com (S.-Y.J.); 2 Developing Product Quality Innovation Team, LG Display, Paju 10845, Republic of Korea; 3 SN Bioscience, 422-Na, LH Business Growth Center, 54 Changeop-ro, Sujeong-gu, Seongnam-si 13449, Republic of Korea

**Keywords:** metal oxides, phase transformation, porosity, tetrahedral aluminum, Lewis sites, acidity

## Abstract

Metal oxides (MOs) having Mg and Al with Mg/Al ratios of 1, 2, 3, and 4 were synthesized via calcination of the layered double hydroxides (LDH). The X-ray diffraction analysis revealed that all the MO consisted of periclase (MgO) crystallite with comparable crystallinity regardless of the metal ratio. According to the ^27^Al magic-angle spinning nuclear magnetic resonance, the phase transformation from LDH to MO upon calcination facilitated the evolution of the Al^3+^ ions with unsaturated coordination at the surface of MO. The specific surface area values of MOs were not significantly different from each other, ranging between 100 and 200 m^2^/g, suggesting that the metal ratio did not strongly influence the porous structure of MO. The temperature-dependent desorption of ammonia demonstrated that the Lewis acidity of the Al-rich MOs was the largest with an Mg/Al ratio of 1, attributed to the efficient exposure of the surface-active site Al^3+^-O^2−^ pairs. The acidity of heterogenous Al-rich MOs significantly increased with the exposed tetrahedral Al site on the surface and dramatically diminished when the molar ratio (Mg/Al) was over two.

## 1. Introduction

Metal oxides (MOs) have attracted increasing attention in various areas such as catalysis, adsorption, and electronics due to their distinctive properties, such as huge specific surface area, tunable porosity, carrier conductivity, and acidity as well as basicity [1,2,3]. Various MOs with different compositions and grain sizes have been developed and utilized in most industrial chemical reactions as catalysts [4]. In addition to the MO containing a single metal species, those with more than two metal species—mixed metal oxides—are widely studied in the academic field due to its structural stability, the coexistence of acid–base sites, and tunable redox properties. Thus, the research on mixed metal oxides lies in a wide spectrum of application fields such as catalysis, adsorption, organic synthesis, green chemistry, petroleum, the pharmaceutical industry, etc. [5,6,7,8,9,10].

Several synthesis methodologies are utilized for the preparation of MOs including layer deposition, hydrothermal method, sol-gel process, ceramic method, and the calcination of metal hydroxides [1,11,12]. Different from other methods, the calcination of metal hydroxides can produce the homogeneous distribution of metal species, and this synthetic route is considered an advanced way to control the physicochemical properties of MO. It has been known that a metal hydroxide with various metal combinations, namely layered double hydroxide or LDH, transforms to MO during the calcination at moderate temperatures (300–600 °C). It forms intimately associated oxide grains with various chemical compositions and a high specific surface area [13]. As the LDH is composed of a mixed metal hydroxide layer with a positive charge (M^2+^_1−x_M^3+^_x_(OH)_2_^x+^) and charge-compensating anions, thermal treatment evaporates the structural hydroxide and anion species to leave only metal oxide crystallites which are interconnected to each other [14]. In addition to the global structural changes, local crystal deformation of the metal hydroxide layers gives rise to coordination disorder around a metal center; this phenomenon is mediated by the partial migration of the trivalent ion—usually Al^3+^ in the MgAl-CO_3_-LDH—from an octahedral site (Oh) to a tetrahedral site (Td), acting as a bridging component between metal oxide crystallites [15].

The MO derived from the precursor LDH has been reported to show catalytic activity concerning the metal combination, M^2+^/M^3+^ ratios, calcination conditions, etc. [16,17]. Several studies reported that MO catalysts can be applied in industrial reactions such as the polymerization of propylene oxide, aldol condensation of acetaldehyde, cycloaddition of CO_2_ to epoxides, and alkylation of aromatic compounds [18,19,20,21]. In addition, Pd-impregnated ZnAl-MO was employed as an acidic catalyst in hydrogenation reactions with superior stability and selectivity to alcohols [22]. We have previously reported that the ZnFe-MO obtained from ZnFe-LDH showed a different catalytic acidity and magnetic properties depending on calcination temperature [23]. The catalytic activity of the MOs primarily relies on the chemical composition, structural properties, and the number of active catalytic sites. In terms of catalytic performance, these oxides generally exhibited acidic behavior as a result of Lewis or Brønsted acidic sites. The Lewis acidic sites can be induced in LDH-derived MOs via tetrahedral coordination of trivalent metal, resulting in catalytically active sites. In this regard, the catalytic performance of LDH-derived MO seems to be dependent on the amount of aluminum.

At this stage, we have encountered two questions: (i) do all Al^3+^ in pristine LDH turn into the Al-Td site for acidity? and (ii) are the Al-Td sites that evolve during calcination well-exposed outside to act as an active site? These questions raised the point that a simple enhancement of Al content in pristine LDH may not guarantee the increase in acid sites in the calcined MO. Despite the extensive utilization of calcination to prepare MO from LDH, to the best of our knowledge, there has been little systematic approaches to elucidate how the active acidic site can be maximized in terms of coordination and surface accessibility in MOs.

**Figure 1:**
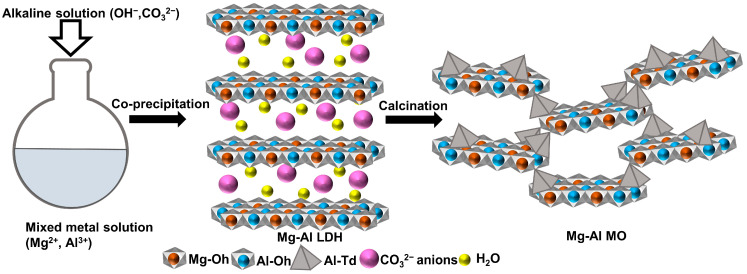
Schematic illustration of MO synthesis.

Herein, we are going to demonstrate a strategy to solve the above problems by optimizing the chemical composition of MO. It will be investigated whether the increase in the amount of trivalent metal, Al^3+^, led to the increase in the surface acidity. In this regard, we have synthesized four MO with different Mg/Al molar ratios via the calcination of MgAl-LDH with the corresponding metal ratios (Figure 1). The chemical composition and structural phase transformation were investigated using an inductively coupled plasma optical emission spectrometer (ICP-OES) and X-ray diffraction (XRD). The coordination environment around Al in MO was elucidated via ^27^Al magic-angle spinning nuclear magnetic resonance spectroscopy (MAS-NMR) and correlated with the surface acidity. The specific surface area and pore volume of the MOs were also measured through N_2_ adsorption–desorption isotherms. Hereafter, the total acidity of the MO samples was estimated from the temperature-programmed desorption of NH_3_, and the optimum synthetic condition for the potential catalytic function will be discussed.

## 2. Results and Discussion

### 2.1. X-Ray Diffraction (XRD) Patterns

The structure of MO with Mg/Al ratios of 1, 2, 3, and 4 (MO-1, MO-2, MO-3, and MO-4, respectively) were investigated in terms of structure and nanoscopic particle arrangement. Before the characterization of MO, we investigated the structural and particulate properties of the precursor LDH in order to comprehend the phase transformation from LDH to MO. The XRD patterns of the pristine LDHs showed a typical hydrotalcite phase (JCPDS No. 14-191) without any significant impurity (Figure S1), regardless of the metal ratio. The FT-IR spectra showed characteristic peaks of hydroxyl, carbonate, and M-O bonds at 3350 cm^−1^, 1390 cm^−1^, and 550 cm^−1^, respectively, revealing that the precursors are only composed of inorganic moieties (Figure S2) [24]. The surface charge of the all the LDHs was found to be highly positive, showing 13.89, 15.22, 18.33, and 13.62 mV for LDHs with Mg/Al ratios of 1, 2, 3, and 4, respectively (Figure S3). It was noteworthy that the ratio between divalent and trivalent metal did not seriously influence the surface positive charge. The hydrodynamic sizes were 1216, 1418, 1595, and 1554 nm with a fairly large polydispersity index, exhibiting that the precursor LDH particles formed agglomerates with a large particle size range (Figure S4).

**Figure 2:**
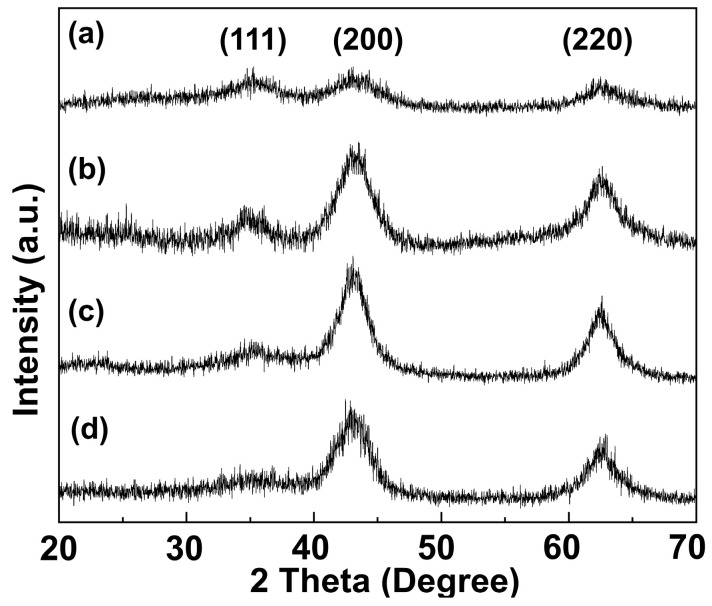
XRD patterns of metal oxides with different Mg/Al ratios: (**a**) MO-1, (**b**) MO-2, (**c**) MO-3, and (**d**) MO-4.

It was confirmed from the XRD patterns (Figure 2) that the four MO had the same crystal phase of periclase (MgO, JCPDS No. 71-1176), with a slight difference in crystallinity depending on the Mg/Al ratios. The diffractograms showed distinctive peaks at 2θ values of 35.04°, 43.07°, and 62.44° corresponding to (111), (200), and (220) planes of periclase, respectively. As previously reported [25], MgAl-CO_3_-LDH underwent dehydration, dehydroxylation, and decarbonization upon heat treatment resulting in the evolution of mixed metal oxide. During the course, most of the Mg^2+^ cations maintained their coordination site in the octahedral center; on the other hand, the Al^3+^ cations underwent partial preservation of octahedral coordination and partial migration to the tetrahedral site [15]. In this way, the major building block of MO would be MgO rather than Al_2_O_3_. As expected, we could not observe any peak corresponding to Al_2_O_3_ even for the MO-1 having the highest Al content. The lattice parameter of all four MO showed the same value of *a* = 4.19 Å. The results revealed that the Mg/Al ratio in the starting LDH did not affect the phase transformation from LDH to the metal oxide through calcination.

Although the four MOs had the same phase, the detailed investigation exhibited differences in crystallinity according to the Mg/Al ratios. It was found that MO-1 had a lower peak intensity (125 count per second (cps) for (200 peak)) than the others (275, 352, and 254 cps for MO-2, MO-3, and MO-4 for (200) peak), suggesting its low crystallinity. The crystallite size of the MOs was calculated based on Scherrer’s equation. As Scherrer’s equation can be applied to the spherical particles, the value itself is not same with the apparent crystallite size. However, we could qualitatively compare the crystallinity difference among samples utilizing Sherrer’s equation. The crystallite size along (200) directions were 20.3, 27.5, 27.5, and 25.1 Å for MO-1, MO-2, MO-3, and MO-4, respectively, showing 25% lower crystallite size of MO-1 than the others. This would be attributed to the low crystallinity of the starting LDH (Figure S1) and the low quantity of Mg for MgO formation. The intensity ratio between the (111) and (200) planes in MO-1 were lower than the other MOs, attributed to the limited crystal growth along (200) due to the lower MgO content in MO-1. The low crystallinity of MO-1 could also be explained by the structural role of the trivalent cation. As denoted in the previous literature [26], LDHs with an M^2+^/M^3+^ ratio ranging from 1.5 to 4 is only reliable in structure. Below the lower limit (M^2+^/M^3+^ < 1.5), the electrostatic repulsion between the adjacent trivalent cations in the lattice reduces the stability, resulting in a less reliable structure of MO-1.

### 2.2. ^27^Al NMR Spectroscopy Analysis

**Figure 3:**
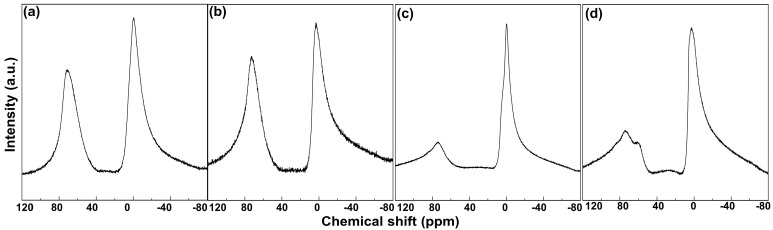
Solid state ^27^Al MAS-NMR spectra of (**a**) MO-1, (**b**) MO-2, (**c**) MO-3, and (**d**) MO-4.

In order to check the chemical composition and local structure, the ICP-OES and the ^27^Al NMR were measured. Since the nominal ratio of Mg/Al and titration range was controlled in the synthesis step, the final molar ratio of Mg/Al in the MO series is various. The ratios were measured as 1.02, 2.01, 2.98, 4.14, which corresponded to MO-1, MO-2, MO-3, MO-4, respectively. Figure 3 shows the ^27^Al NMR spectra of the MO samples in which the peaks formed at around 0 ppm were assigned to the Al ions occupying the octahedral sites (Al-Oh), and those at around 70 ppm were attributed to the tetrahedral coordination (Al-Td), respectively [27,28]. As described in the previous reports, parent LDHs underwent a phase transformation to MO through the partial migration of Al^3+^ from Oh to Td sites, leading to the crystal deformation of the layers [15,29,30,31].

The nuclear spin of Al is strongly affected by a variety of interactions, including chemical shielding, dipole–dipole, or quadrupole interactions. Chemical shielding, which is determined by the surrounding environment of the Al nucleus affecting the chemical shift. The more the Al is surrounded by O ligands, the more electron deficient the Al nucleus becomes, and the chemical shift appears in downfield. Empirically, the chemical shift in Al-NMR was highly proportional to the coordination number; coordination numbers 4, 5, and 6 in aluminum hydroxide or oxide appeared in ~70 ppm, ~35 ppm, and ~0 ppm, respectively [27]. Furthermore, the spectral lines of Al-NMR can be broadened depending on the surroundings of the nucleus [28]. The coexistence of two peaks at 0 and 70 ppm suggested the partial preservation of Al-Oh and partial migration into Al-Td. It was noted that the Al-rich samples—MO-1 and MO-2—showed relatively well-developed Al-Td peaks compared with the Al-deficient ones, MO-3 and MO-4. We could reason that the Oh-to-Td migration of Al^3+^ and the subsequent disorders [29] were more facilitated in the Al-rich LDH, where the threshold Mg/Al ratio lay between 2 and 3. The discrepancy in the local disorder may be addressed by the stability of the parent LDH; since the Mg/Al molar ratio of 3 is naturally found in the form of hydrotalcite [30], the structure may be more resistant to the phase transformation or metal migration.

The Al-Oh peak in MO-3 was found to be the sharpest in terms of full-width-at-half-maximum (17.73, 17.33, 12.91, and 16.23 ppm for the Al-Oh peaks of MO-1, MO-2, MO-3, and MO-4). Figure 3c also shows the presence of a shoulder peak around 60 ppm, which is also attributed to the AlO_4_ according to the previous report [27] (deconvoluted spectra are shown in Figure S6). As the Td site is more favorable than the Oh site in terms of additional coordination, the disordered structures found in MO-1 and MO-2 may be advantageous compared with MO-3 and MO-4. Intriguingly, the spectrum of MO-4 was slightly different from the others, showing a shoulder Al-Td peak. This phenomenon was attributed to the presence of slightly different Al environments formed by the random distribution of Al in the LDH layers of Mg/Al ratio 4 [26]. As the randomness of Al is not a controllable factor, we could not expect that MO-4 would be a good candidate for a catalyst.

Quantitative analyses for ^27^Al-NMR spectra were performed to estimate how much of the Al in LDH migrated into the Td site during calcination and to estimate the degree of disorder around Al in MO (Table 1). As the coordination number and environment have vital roles in the acidity of the MgAl-MO, the peaks in NMR were separated based on Gaussian functions. We analyzed the portion of the Td and Oh sites by calculating the ratio between Al-Td and Al-Oh. The calculated peak ratios were 0.81, 0.88, 0.40, and 0.35 for MO-1, MO-2, MO-3, and MO-4, respectively, showing a very high portion of tetrahedral AlO_4_ units over the octahedral AlO_6_ units in MO-1 and MO-2. According to the previous literature, the transitional alumina obtained via thermal treatment of bayerite or boehmite showed a Al-Td/Al-Oh ratio of 0.3–0.4 [31]. The paper claimed that the Al-Td/Al-Oh ratio was fairly high due to both the high specific surface area and high amount of surface-exposed Al. Andrew et al. reported that calcination of synthetic LDH resulted in a high Al-Td/Al-Oh ratio of 0.4 [32]. They found that the Mg/Al ratio at the surface and bulk showed different Al concentrations. Based on the previous literature, we could infer that the calcination of MgAl-LDH efficiently exposed the Al moiety to the surface and those surface Al is usually located in Td sites. Considering this hypothesis, we could expect the location and characteristic of Al in the Mos depending on the Mg/Al ratios. From Table 1, the Al-Td/Al-Oh was fairly proportional to the Al composition in the precursor LDH. In other words, the more Al exists in the precursor LDH, the higher portion of Al is exposed to the surface in the Td coordinate. As the surface-exposed tetrahedra Al practically acts as an acidic site, the results suggested that the higher composition of Al in the precursor LDH is recommended for the MO with acidic sites. In addition to the peak area ratio, the peak position is also worthy to note.

**Figure 4:**
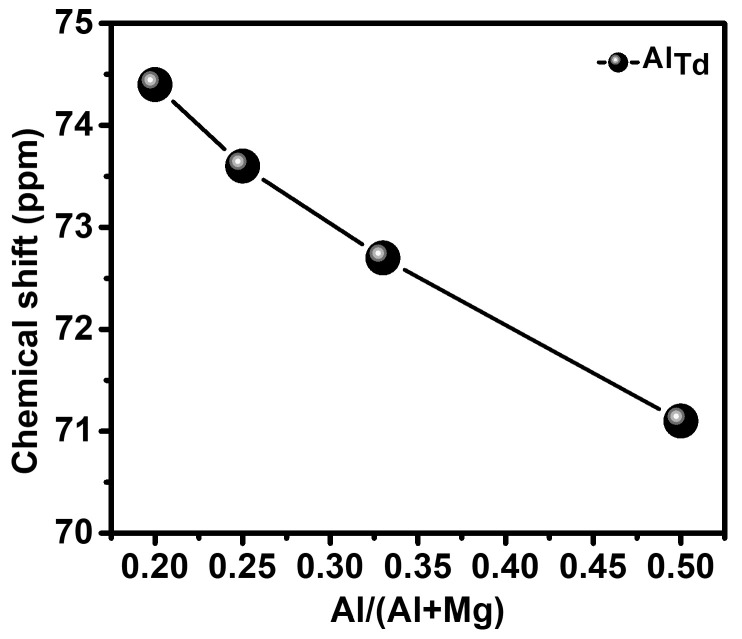
^27^Al NMR analysis of the MO samples. Effect of chemical composition on the chemical shift values of Al-Td.

As displayed in Table 1, the peak position of the Al-Td moved towards a positive direction with decreasing aluminum content. This phenomenon can be explained on the basis of the condensation effect of AlO_4_ and electronegativity of Al. As previously reported [33], the chemical shift of the Al-Td is associated with the degree of condensation; a high chemical shift correlated with a low degree of condensation [34]. The Al-Td peak of both MO-1 and MO-2 appeared in the lower chemical shift region than MO-3 and MO-4, indicating the higher condensation of AlO_4_ tetrahedra in Al-rich Mos. We could suggest a mechanism as follows for MO-1; the Al migrated from octahedral to tetrahedral sites during calcination and the Al-Td moieties condensed to form surface-exposed AlO_4_. It is worthy to note here that the chemical shift for Al-Td gradually decreased as a function of the Al content in a linear manner, as shown in Figure 4. This chemical shift was attributed to the gradual replacement of Mg^2+^ by the more electronegative Al^3+^ as the nearest neighbor cation which was bonded to the Al-O polyhedral [35,36]. The Al-Td (70.6 ppm) peak of MO-1 observed in the low chemical shift suggested that the Al was sufficiently surrounded by the AlO_4_ moiety. In other words, the more Al exist in the pristine LDH, the more Al-Td could be exposed at the surface of MO [36].

### 2.3. N_2_ Adsorption–Desorption Isotherms

**Figure 5:**
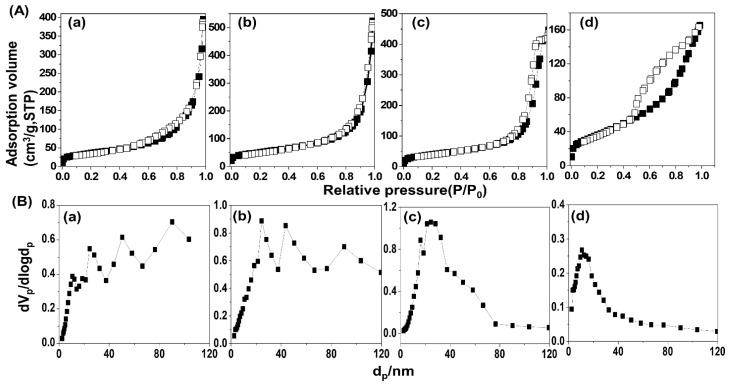
(**A**). N_2_ adsorption and desorption and corresponding (closed symbols: adsorption, open symbols: desorption) (**B**) BJH pore size distribution plot: (**a**) MO-1, (**b**) MO-2, (**c**) MO-3, and (**d**) MO-4.

As the porosity and specific surface area are crucial parameters for metal oxide catalysts, we obtained N_2_ adsorption–desorption isotherms for detailed analyses. The isotherm curves of all the MO (Figure 5A) exhibited type II adsorption according to the International Union of Pure and Applied Chemistry (IUPAC) classification [37]. Type II adsorption is observed when the material has a wide spectrum of pore size [38,39], suggesting the evolution of various pores in MO during calcination of LDH.

As previously reported [29,40], LDH undergoes sequential gas release to form an oxide with both interparticle and intraparticle pores. In this manner, the MO obtained by calcination of LDH tends to have both small intraparticle pore and interparticle pores, i.e., a wide range of pore size is shown in Figure 5B. Similar adsorption types and wide pore size distribution were often reported in other kinds of calcined LDH due to the evolution of porous structures. The surface morphology of the MO was investigated with SEM and is displayed in Figure S5. All the MO, regardless of the metal ratio, showed large lumps which were made up of small particles. Although it is not clear whether the small grains were MO or MgO nanoparticles, from the SEM images, we could confirm that the small MgO nanoparticles were gathered to form large particles rather than separated particle-by-particle. Therefore, the pore structure observed in the N_2_ adsorption–desorption isotherms was attributed to both the intraparticle and interparticle pores. We could observe slight hysteresis in the isotherm of MO-3 in the high relative pressure, which was attributed to the capillary condensation resulting from the interparticle pore [41,42,43]. It was notable that MO-4 had distinctive H3 hysteresis according to the IUPAC classification [37]. This hysteresis is observed when the materials have slit-like pores. During the degassing of LDH upon calcination, there evolved small MgO domains which interstratified through the tetrahedral AlO_4_ units [44] As MO-4 had the largest quantity of Mg in the pristine LDH, they might develop large MgO domains with plate-like shapes, resulting in the slit-like pores.

It should be noted that the specific surface area of all four MOs were comparable regardless of the differences in pore structures. The specific surface area values investigated by the Brunauer–Emmett–Teller (BET) methods were 124.7, 173.8, 139.78, and 134.65 m^2^/g, respectively, for MO-1, MO-2, MO-3, and MO-4 (Table 2). It has been generally reported that LDH having less than 100 m^2^/g S_BET_ (specific surface area calculated via the BET method) transformed to MO with S_BET_ between 100 and 200 m^2^/g. According to our previous study [45], the specific surface area of LDH-derived MO tended to higher when the M^2+^/M^3+^ ratios were within a moderate range. This would be related to the structural stability of pristine LDH depending on the metal ratio [26]. We could also observe that the specific surface area was slightly higher in MO-2 than the others might be due to the higher Al-Td content. The two MO with moderate Mg/Al ratios (MO-2 and MO-3) had homogeneous pore structure in terms of the pore size distribution (Figure 5B) and pore volume (Table 2). Therefore, we could temporarily conclude that the moderate Mg/Al ratios would be favorable in the development of MO-based adsorbents.

### 2.4. NH_3_-Temperature-Programmed Desorption

**Figure 6:**
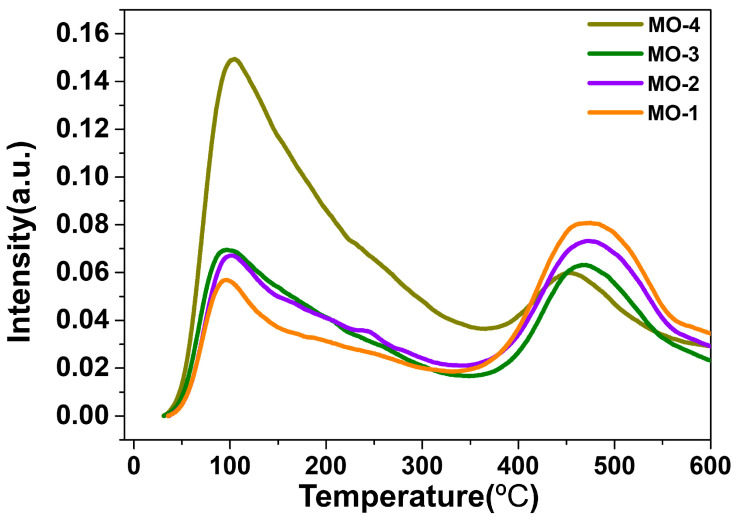
NH_3_-TPD profile of MO-1, MO-2, MO-3, and MO-4.

The acidity of the four MO samples was estimated via NH_3_-TPD as shown in Figure 6. The desorption peaks formed at a low-temperature (60–150 °C) range were attributed to the physically adsorbed ammonia at the weak acidic sites of the MO samples, whereas the peaks observed at a higher temperature (400–600 °C) range resulted from the desorption of NH_3_ molecules at the strong acidic sites [46,47].

**Figure 7:**
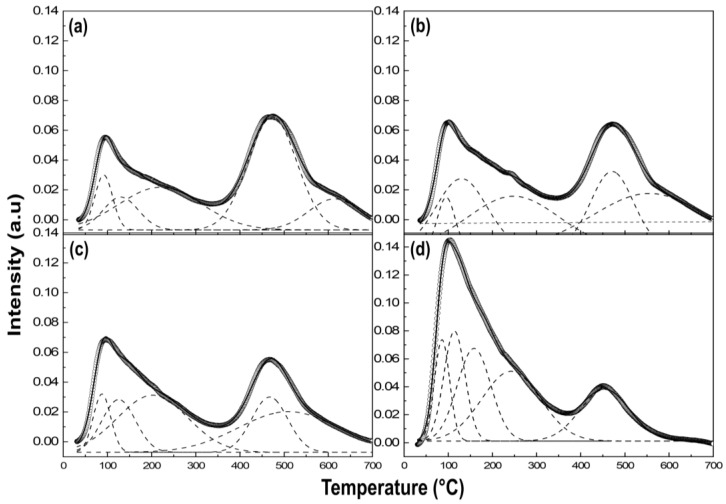
Deconvoluted NH_3_-TPD spectrum: (**a**) MO-1, (**b**) MO-2, (**c**) MO-3, and (**d**) MO-4 (solid line: observed data; open circles: summation of separated peaks; dotted lines: each peak components).

In order to account for the total distribution of acidic strength of MOs, since the Mg/Al ratio considerably alters the number of acidic sites in the MO samples, the curves were separated into several distinguishable components (Figure 7). The solid lines represent the experimental data, and the dotted lines correspond to the individual component. The acid strength of MOs corresponds to the peak maxima obtained at higher desorption temperatures of the NH_3_ molecules [48]. It was noted that MO-1 displayed the strongest acidity among the four Mos, which were confirmed by the two large peaks at high temperatures of 614.2 °C and 473.6 °C (Figure 7a). By contrast, MO-2 displayed high temperature peaks at 470.7 °C and 555 °C (Figure 7b). This is due to the existence of the surface-exposed and unsaturated Al at the Lewis acidic sites [23]. The low-temperature peaks obtained between 80 and 250 °C for the MOs were attributed to the weak Bronsted acidic sites resulting from the surface hydroxyl groups. The desorption peak area of the high temperature region of the MOs increased with Al content, indicating that Al^3+^ provides the most active sites to act as a catalyst and the catalyst surface is dominated by the Lewis acid sites [49]. In general, the strong acid site originated from Al and the weak acid site is attributed to both Al (Lewis acid) and Mg-OH (Brønsted acid). The tetrahedral Al sites led to the exposure of acidic sites to enhance strong acidity in the samples [50,51]. In the resolved spectra, MO-3 showed three component bands at lower temperatures and two high-temperature desorption peaks at 467.7 °C and 509.9 °C (Figure 7c), whereas MO-4 showed four component bands at low temperatures and only one peak maximum at 454.6 °C, indicating that the weak acidic sites were predominant in MO-4 (Figure 7d), which refers to the lower catalytic acidity. It was found that MO-4 had the least Al-Td portion and low Al content and exhibited an intense desorption peak at low temperatures and a relatively small peak at the high-temperature range, indicating that the acidic Al-Td sites were screened by the MgO moiety [52].The Al-Td content in MOs shows a correlation that as the Al-Td content increases the surface acidity of the MOs also increases.

The number of acid sites was quantitatively estimated from the integrated peak area of the NH_3_-TPD curves, which is summarized in Table 3. The four MOs were divided into two groups in terms of the number of acidic sites: MO-1 and MO-2 with high acid sites, and MO-3 and MO-4 with low acid sites. The acidic strength of the MO samples could also be explained in terms of the existence of Al-Td; therefore, we compared the number of tetrahedral Al sites in each MO by multiplying the Al-Td site (STd: peak area for Al-Td in NMR) and Al content in wt% (Table 3).

The estimated values indicate that acidic strength of MO-1 was slightly higher than MO-2, while MO-3 and MO-4 were low in acidic strength. This should be correlated to the abundant Al^3+^-O^2−^ pair on the surface of MO-1 and MO-2 acting as active sites [35]. Thus the enhanced acidic strength of the Al-rich MOs was primarily associated with the formation of surface-exposed tetrahedrally-coordinated aluminum (i.e., AlO_4_) [53]. The correlation between the surface acidity of the MO samples with the Mg/Al molar ratio demonstrates that the total acidity was increasing with the decreasing Mg/Al ratio due to Lewis’s acidic sites induced by the coordinatively unsaturated Al-Td. In terms of the number of acidic sites, the MO samples can be arranged in the following order: MO-1 ≈ MO-2 >> MO-3 ≈ MO-4. In addition, to the strength of acidity, the MO can be ordered as follows: MO-1 > MO-2 > MO-3 >> MO-4.

## 3. Materials and Methods

Magnesium nitrate hexahydrate (Mg(NO_3_)_2_·6H_2_O) and aluminum nitrate nonahydrate (Al(NO_3_)_3_·9H_2_O) were purchased from Sigma-Aldrich, LLC. (Burlington, MA, USA). Sodium hydroxide (NaOH) and sodium bicarbonate (NaHCO_3_) were obtained from Daejung Chemicals & Metal Co., Ltd. (Siheung-si, Republic of Korea).

The powder XRD patterns of MO were analyzed via an X-ray diffractometer (Ultima IV, Rigaku, Tokyo, Japan) using Cu Kα radiation (*λ* = 1.5405 Å) at 50 kV and 40 mA in the range of 2*θ* = 5–80° with an interval of 0.02° and a scan speed of 0.5°/min. The lattice parameter was calculated using UnitCell software 2006 (Tim Holland & Simon Redfern, 2006, Cambridge University, Cambridge, UK) based on the X-ray diffractograms. The crystallite size of the samples along a specific crystal plane was calculated by using Scherrer’s equation [54] as shown below:

*τ* = (0.9*λ*)/(B cos *θ*)

where *τ* = crystallite size (Å), *λ* = X-ray wavelength (1.5405 Å), B = full-width at half-maximum (FWHM), and *θ* = Bragg’s angle.

The N_2_ adsorption–desorption isotherms were measured via BEL-SORP-mini II (Microtac BEL, Inc., Tokyo, Japan) at 77 K to analyze the specific surface area and pore size distribution. All four samples were degassed at 423 K in a vacuum condition overnight before the isotherm measurement. The specific surface area was calculated based on the Brunauer–Emmett–Teller (BET) theory. The pore size distribution was determined using the Barett–Joyner–Halenda (BJH) model. The surface morphology of the particles was analyzed via scanning electron microscopy (SEM, JEOL-7100F, Tokyo, Japan) at an acceleration voltage of 15 kV. The powder sample was spread on the carbon tape and blown away using a silicon air blower. The specimen was coated with Pt/Pd via vacuum sputtering for 2 min. Fourier transformed infrared (FT-IR) spectra were recorded on a FT/IR-4600 spectrometer (JASCO, Tokyo, Japan) over a range of (4000–500) cm^−1^ with a 4 cm^−1^ resolution to detect transmission at room temperature. All the four LDHs powders were placed on the crystal and directly recorded via ATR mode. The particle size and the zeta-potential of the LDHs were evaluated via a Zeta potential-particle size analyzer of ELSZ-100 (Otsuka Electronics Co., Ltd., Osaka, Japan).

The contents of Mg^2+^ and Al^3+^ present in the MO samples was measured using ICP-OES (NexION 2000, Perkin Elmer, Middlesex, MA, USA). The samples were prepared by digesting 10 mg of the MO in 10 mL of concentrated HCl and diluted to 50 mL with deionized water. Solid-state ^27^Al-NMR (Unity INOVA, Agilent Technologies, Santa Clara, CA, USA) was obtained at a Larmor frequency of 156.320 MHz through the Korea Basic Science Institute (KBSI) Western Seoul Center. The chemical shift was represented in ppm from AlCl_3_, and the measurement repetition time was 2 s during the analysis. The spectrum was recorded in a 1/12 π pulse (0.83 μs), with a spinning rate of 22 kHz. The total amount of acid sites and their strengths in MO were evaluated via utilizing TPD in a Catalyst Analyzer BELCAT-M (Microtrac BEL, Corp., Osaka, Japan). Ammonia was used as the probe molecule and the MO samples (50 mg) were preheated at 100 °C for 1 h under He gas flow. After NH_3_ sorption for 10 min at room temperature using NH_3_/He mixed gas flow, the sample was refreshed for another 10 min under He, followed by increasing the temperature up to 800 °C with a heating rate of 10 °C/min.

Pristine LDH with various Mg/Al molar ratios was synthesized via the conventional co-precipitation method [55]. The mixed metal solutions with Mg/Al ratios of 1, 2, 3, and 4 were prepared while the total metal concentration was set to 0.045 mol/L. The alkaline solution was prepared by dissolving 0.208 mol of NaOH and 0.28 mol of NaHCO_3_ in 500 mL of deionized water. The mixed metal solutions were titrated with an alkaline solution in a reactor by keeping pH~9.5. After 24 h of reaction at room temperature, the white suspension was centrifuged and washed thoroughly with deionized water three times. Finally, the precipitate was lyophilized to obtain the powdery sample. Then, the LDH powder was calcined at 400 °C for 9 h with a 0.8 °C/min of heating rate in a muffle furnace. The obtained metal oxide with starting Mg/Al ratios of 1, 2, 3, and 4 were named MO-1, MO-2, MO-3, and MO-4, respectively.

## 4. Conclusions

We have successfully synthesized Mg-Al binary oxides with the Mg/Al ratios of 1, 2, 3, and 4 by the calcination of LDHs precursors. Calcination results in the formation of the MgO phase for all MOs, with low crystallinity for MO-1. According to ^27^Al NMR analysis, the Al-rich MOs displayed the highest Al-Td fraction by exposing more Al-Td on the surface upon calcination. It was found that MO-1 contains relatively more Al-Td-O tetrahedra connected with the Al^3+^ ions. The specific surface area and pore volume of MO-2 were higher than the others. We have confirmed that the acidity of the Al-rich MO-1 was slightly superior to MO-2 due to the strong Lewis acidic sites which were driven by the solid phase transformation, leading to the evolution of more tetrahedrally-coordinated and unsaturated Al. The Al-rich MOs replaced more Mg^2+^, resulting in the formation of heterogeneous surface-enriched Al^3+-^O^2−^ active catalytic sites. The controlled incorporation of trivalent Al^3+^ is advantageous to producing structurally heterogenous Mg-Al mixed oxides derived from LDHs to obtain more surface-active catalysts and catalytic supports.

## Figures and Tables

**Table 1:** Peak analyses result of the MO samples from solid state ^27^Al NMR spectra (Al wt% was calculated from ICP-OES).

Sample	Peak Position (ppm)		Area Ratio (Al-Td/Al-Oh)	Al wt% in MO
	Al-Td	Al-Oh		
MO-1	71.1	−0.57	0.81	24.3
MO-2	72.7	3.1	0.88	16.4
MO-3	73.6	−0.4	0.40	12.5
MO-4	74.4	2.9	0.35	9.5

**Table 2:** Porosity information of MO-1, MO-2, MO-3, and MO-4.

Parameter	MO-1	MO-2	MO-3	MO-4
S_BET_ (m^2^/g)	124.7	173.8	139.78	134.65
Pore volume (cm^3^/g)	0.61	0.81	0.66	0.26
Mean pore diameter (nm)	19.57	18.65	18.93	7.60

**Table 3:** Data from the ^27^Al NMR spectra and NH_3_-TPD measurement of MO-1, MO-2, MO-3, and MO-4 samples. *STd: Area for Al-Td site in Al-NMR.

Sample	*STd × Al wt. %	The Area under High Temperature	The Area under Low Temperature
MO-1	10.88	11.18	8.65
MO-2	7.69	10.3	10.8
MO-3	3.60	4.23	14.67
MO-4	2.47	4.84	21.4

## Data Availability

All data generated or analyzed during this study are included in this article.

## Supplementary Materials

The following supporting information can be downloaded at: https://www.mdpi.com/article/10.3390/molecules28166072/s1, Figure S1: XRD patterns for (a) LDH-1, (b) LDH-2, (C) LDH-3, and (d) LDH-4; Figure S2: FT-IR spectra for (a) LDH-1, (b) LDH-2, (c) LDH-3, and (d) LDH-4; Figure S3: Zeta potential for LDH-1, LDH-2, LDH-3 and LDH-4; Figure S4: DLS for LDH-1, LDH-2, LDH-3 and LDH-4; Figure S5: Scanning electron microscopy images (SEM) for (a) LDH-1, (b) LDH-2, (c) LDH-3, (d) LDH-4; Figure S6: Deconvoluted solid-state ^27^Al MAS-spectra for (a) MO-3, and (b) MO-4.
