# Supplementary material for: Effect of Tetrahedrally Coordinated Al on the Surface Acidity of Mg-Al Binary Mixed Oxides
Source: Molecules. 2023 Aug 15;28(16):6072. doi: 10.3390/molecules28166072 (PMC10459910; doi:10.3390/molecules28166072)
Supplement: Supplementary file 1 [file molecules-28-06072-s001.zip › molecules-2508341-supplementary.pdf]

Supplementary Information

Effect of Tetrahedrally Coordinated Al on the Surface Acidity of Mg-Al Binary Mixed Oxides

Vidya Chandrabose [1,†], Taeho Kim [1,†], Ji won Park [1,2], Sang-Yong Jung [1,3] and Jae-Min Oh [1,*]

[1] Department of Energy and Materials Engineering, Dongguk University-Seoul, Seoul 04620, Republic of Korea; cbvidya1997@gmail.com (V.C.); taeho0408@naver.com (T.K.); qkrwldnjs324@naver.com (J.w.P.); syjung05@naver.com (S.-Y.J.)

[2] Developing Product Quality Innovation Team, LG Display, Paju 10845, Republic of Korea

[3] SN Bioscience, 422-Na, LH Business Growth Center, 54 Changeop-ro, Sujeong-gu, Seongnam-si 13449, Republic of Korea

* Correspondence: jaemin.oh@dongguk.edu

† These authors contributed equally to this work.

1. XRD

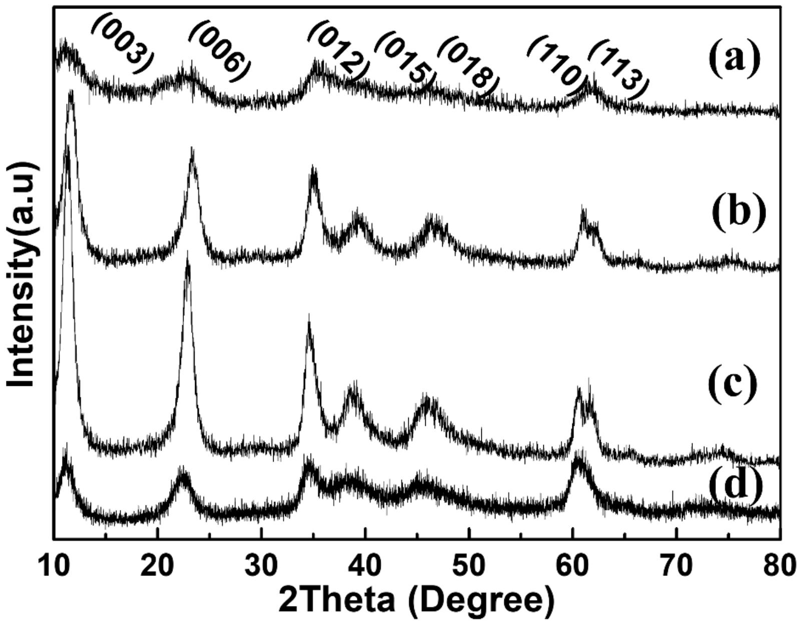

Figure S1. XRD patterns of pristine LDHs (a) LDH-1, (b) LDH-2, (c) LDH-3, and (d) LDH-4.

2. FT-IR

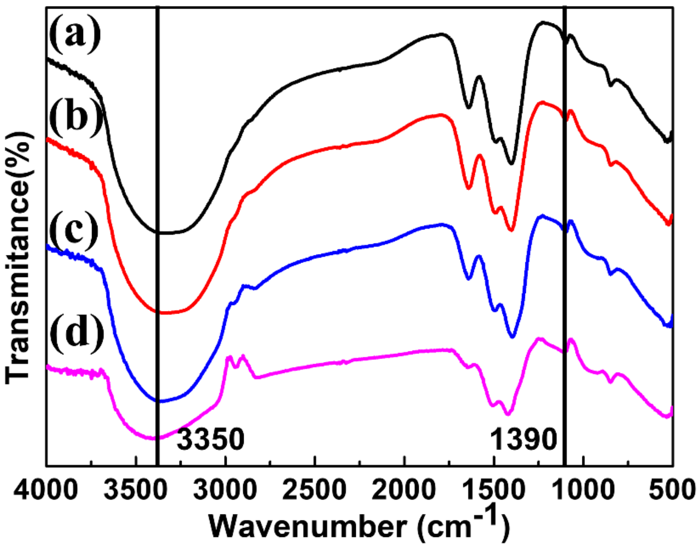

Figure S2. FT-IR spectra of pristine LDHs (a) LDH-1, (b) LDH-2, (c) LDH-3, and (d) LDH-4.

3. Zeta potential

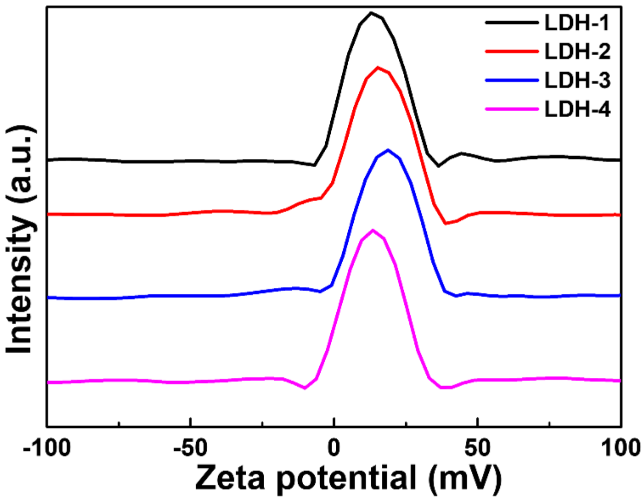

Figure S3. Zeta potential of pristine LDHs: LDH-1, LDH-2, LDH-3, and LDH-4.

4. DLS

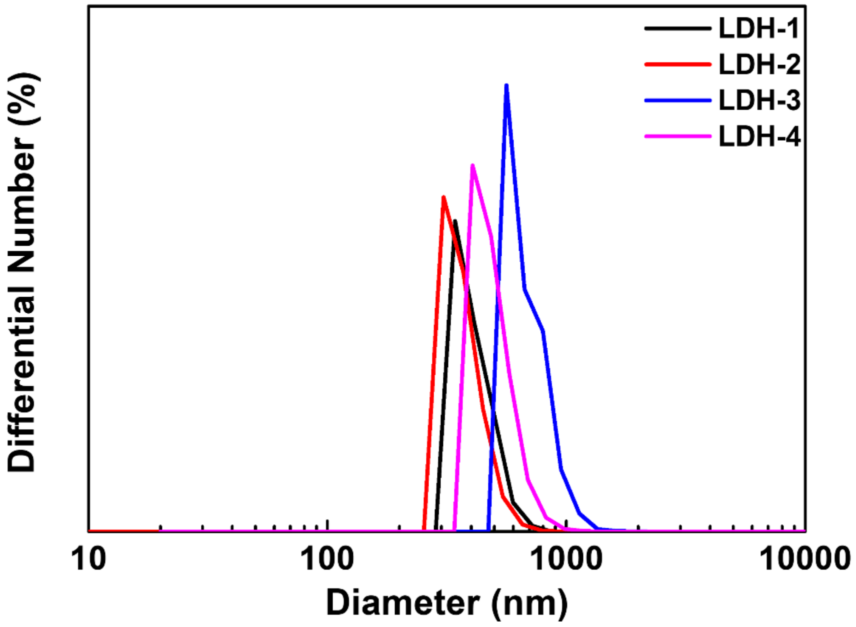

Figure S4. DLS of pristine LDHs: LDH-1, LDH-2, LDH-3, and LDH-4.

5. SEM

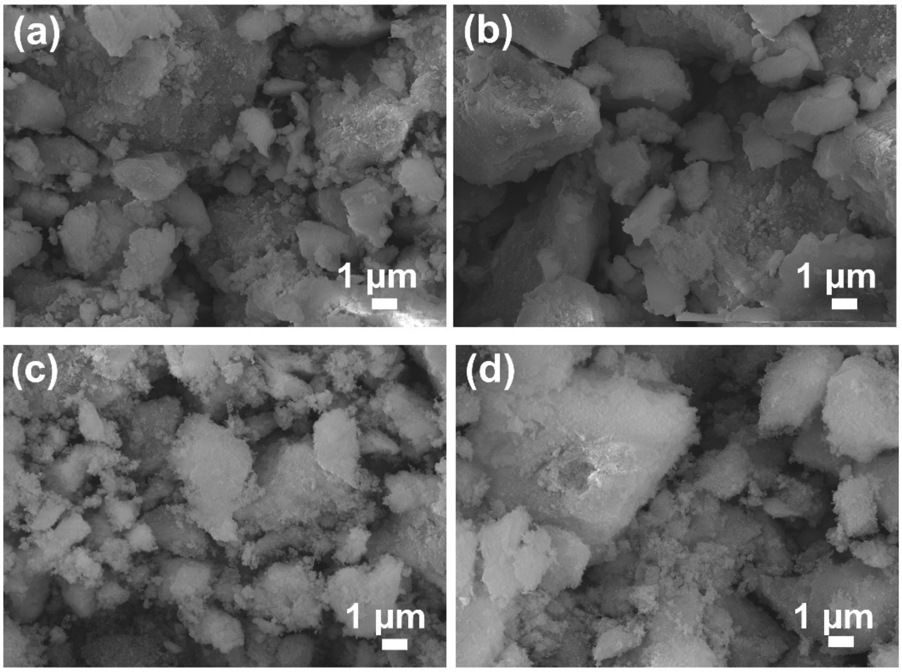

Figure S5. Scanning electron microscopy (SEM) images of (a) MO-1, (b) MO-2, (c) MO-3, and (d) MO-4.

6. ^{27}AlNMR deconvoluted spectra

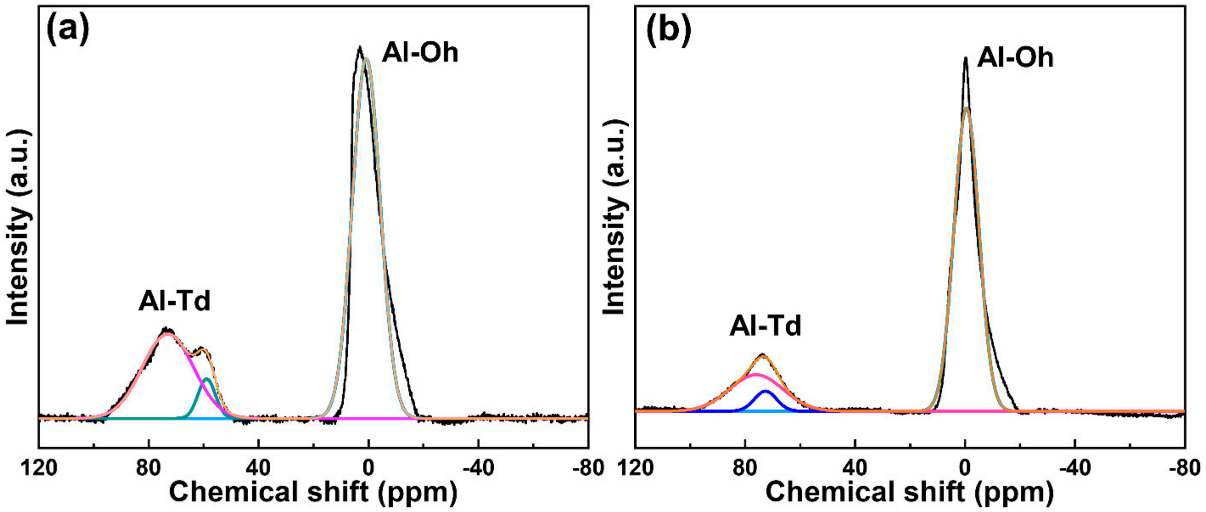

Figure S6. Deconvoluted Solid state ^{27}Al MAS-NMR spectra of (a) MO-3 and (b) MO-4.
